# Understanding the Relationships between Gender Inequitable Behaviours, Childhood Trauma and Socio-Economic Status in Single and Multiple Perpetrator Rape in Rural South Africa: Structural Equation Modelling

**DOI:** 10.1371/journal.pone.0154903

**Published:** 2016-05-16

**Authors:** Rachel Jewkes, Mzikazi Nduna, Nwabisa Jama-Shai, Esnat Chirwa, Kristin Dunkle

**Affiliations:** 1 Gender & Health Research Unit, Medical Research Council, Pretoria, South Africa; 2 School of Public Health, University of the Witwatersrand, Johannesburg, South Africa; 3 Department of Psychology, University of the Witwatersrand, Johannesburg, South Africa; Massachusetts General Hospital, UNITED STATES

## Abstract

**Background:**

Interventions to prevent rape perpetration must be designed to address its drivers. This paper seeks to extend understanding of drivers of single and multiple perpetrator rape (referred to here as SPR and MPR respectively) and the relationships between socio-economic status, childhood trauma, peer pressure, other masculine behaviours and rape.

**Method:**

1370 young men aged 15 to 26 were interviewed as part of the randomised controlled trial evaluation of Stepping Stones in the rural Eastern Cape. We used multinomial to compare the characteristics of men who reported rape perpetration at baseline. We used structural equation modelling (SEM) to examine pathways to rape perpetration.

**Results:**

76.1% of young men had never raped, 10.0% had perpetrated SPR and 13.9% MPR. The factors associated with both MPR and SPR (compared to never having raped) were indicators of socio-economic status (SES), childhood trauma, sexual coercion by a woman, drug and alcohol use, peer pressure susceptibility, having had transactional sex, multiple sexual partners and being physically violent towards a partner. The SEM showed the relationship between SES and rape perpetration to be mediated by gender inequitable masculinity. It was complex as there was a direct path indicating that SES correlated with the masculinity variable directly such that men of higher SES had more gender inequitable masculinities, and indirect path mediated by peer pressure resistance indicated that the former pertained so long as men lacked peer pressure resistance. Having a higher SES conveyed greater resistance for some men. There was also a path mediated through childhood trauma, such that men of lower SES were more likely to have a higher childhood trauma exposure and this correlated with a higher likelihood of having the gender inequitable masculinity (with or without the mediating effect of peer pressure resistance).

**Discussion:**

Both higher and lower socio-economic status were associated with raping. Prevention of rape perpetration must focus on changing men’s gender ideals, entitlements and inequitable practices. Reducing poverty and adverse childhood experiences should also be of benefit.

## Introduction

Rape may be perpetrated by men on their own (single perpetrator rape), or with others (multiple perpetrator rape) and in all populations studied, a fairly high proportion of the adult male population disclose having ever raped (between 11–61%)[1, 2]. Since very few men are jailed for rape each year, prevention among men in the general population is a priority.

There has been research on rape causation, but some areas remain unclear. One of these is the relationship between social marginalisation and rape perpetration. Research thus far shows that in some settings men who have perpetrated multiple perpetrator rape (MPR) are relatively more socially marginalised than other men–with poorer education and more food insecurity [1], whereas in others, including South Africa, a notable finding is that men who rape, including MPR, are wealthier and have relatively educated mothers, which may be indicative of having greater social privilege [2–4].

Research thus far has found a fairly consistent set of male behaviours to be associated with rape perpetration. These include the use of physical intimate partner violence, having higher numbers of sexual partners, and transactional sex [3–6]. These factors have been interpreted as indicating gender inequitable constructions of masculinity, but the empirical basis for this is deserving of further research. Holding gender inequitable attitudes, including viewing women instrumentally, are often also associated with rape perpetration [2, 7, 8].

There is also evidence that men who have experienced trauma in childhood and/or sexual abuse are more likely to rape [1, 7, 9]. Further, rape perpetration is associated with a range of indicators of anti-social behaviour [10, 11]. Population-based research shows that men who have perpetrated rape are more likely to have been gang members, have weapons, fight with other men and have used drugs and abused alcohol [1, 5, 6]. These behaviours may be associated with a context of poverty and male disempowerment [11], and are often interpreted as reflecting a hypermasculinity [12]. However current sociological thinking positions hypermasculinity as an extension of hegemonic masculinity (to use RW Connell’s term), rather than being a ‘different masculinity’ [13] and that it emerges out of the relationship between hegemonic ideals and (some) men’s inability to meet them in contexts of poverty [14]. This has not been yet demonstrated in quantitative sociological research, Effective intervention to prevent rape requires a more nuanced understanding of drivers of rape perpetration, including understanding the relationships between rape perpetrated by a single perpetrator (SPR) and that by multiple perpetrators (MPR) [15]. In recent years more has been written about MPR but until recently it received little specific focus in the rape literature [1, 4, 16–18].

In this paper we use South African data collected during the Stepping Stones trial to extend our knowledge of drivers of rape perpetration and consider the relationship between SPR and MPR. We specifically seek to describe the factors associated with the two types of rape using a multinomial regression model and to use structural equation modelling (SEM) on cross-sectional data to further elucidate the relationships between social marginalisation (socio-economic status), childhood trauma, peer pressure, indicators of gender-inequitable masculinity and rape. We hypothesise that socio-economic status may influence childhood trauma, the ability to resist peer pressure and constructions of masculinity and that all of these may impact rape perpetration.

## Methods

Between 2002–2003 we enrolled 1370 men aged 15–26 years into a cluster randomised controlled trial to evaluate the HIV prevention behavioural intervention Stepping Stones [19]. The trial had two arms. One received Stepping Stones, a 50 hour participatory intervention on sexual health and HIV, delivered over 6–8 weeks. The control arm received one 3 hour session on safer sex and HIV. Otherwise the participants in the two arms were treated no differently. The men were volunteers recruited from 70 locations (clusters) in the Eastern Cape province of South Africa, mainly from schools. The clusters were divided into seven geographically-defined strata. In each cluster, 15–25 male youth were enrolled (women are not discussed this paper). Assessments used a questionnaire administered by age and sex matched interviewers, and most baseline interviews were conducted by two men. Further information on all assessments, study recruitment, access and ethical issues, is presented elsewhere [20, 21]. Details of the main more complex measures used are presented in Table 1. We also asked about age, years of completed schooling, having ever earned money, the level of schooling obtained by their mother, any gang membership and whether he had ever been arrested (for anything). For the first part of the analysis presented, we classified men into three categories: never perpetrated rape, perpetrated single perpetrator rape (SPR) but not MPR, and perpetrated MPR (regardless of SPR). This was in keeping with our hypothesis that MPR is an escalation of SPR [4].

**Table 1:** Measures.

Latent	Indicator	Definition		R 2	Items covarying
**Rape**				0.85	
	Sexual IPV	continuous	4 items about having had physically forced sex, sex when he knew she did not want to but was afraid, and forced oral/anal sex, modified from the World Health Organisation’s instrument (22); Scores ranges 8–23	0.25	
	Multiple perpetrator rape	binary	2 items "Was there ever an occasion when you and other men had sex with a woman against her will or when she was too drunk to stop you?” and “have you ever done streamlining?”; overall prevalence 13.9%	0.17	
	non-partner rape	binary	3 items asking about sex with a woman too drunk to consent or stop it, and having ‘made’ a woman non-partner have sex when she didn’t want to. We also asked about attempted rape; the response was presented as a yes/no and 10.9% said yes	0.25	
**Socio-economic status**		(exogenous)			
	Hunger	4 response options	Would you say that the people in your home often, sometimes, seldom or never go without food?	0.64	with no meat
	No money for meat	4 response options	Would you say that people in your home often, sometimes, seldom or never have a day when they do not eat meat?*	0.5	with emergency resource mobilisation
	Emergency resource mobilisation	4 response options	If a person became ill in your home and R100 was needed for treatment or medicines, would you say it would be very easy, easy, quite difficult or very difficult to find the money?	0.21	
**Childhood trauma**			a modified version of the short form of the Childhood Trauma Questionnaire (23), details presented in Jewkes et al (24)	0.06	
	emotional abuse	continuous	3 items with never, sometimes, often and very often response categories (scores ranged 3–9)	0.41	with physical hardship
	emotional neglect	continuous	3 items with never, sometimes, often and very often response categories (scores ranged 3–12)	0.42	with emotional abuse
	physical abuse	continuous	4 items with never, sometimes, often and very often response categories (score range 4–16)	0.24	with emotional abuse
	sexual abuse	continuous	3 items with never, sometimes, often and very often response categories (score range 2–8)	0.11	
	physical hardship	continuous	4 items with never, sometimes, often and very often response categories (score range 4–16)	0.53	
**Gender inequitable masculinity**				0.18	
	drug use	continuous	score of responses to 5 questions about drug use (range scaled from 0–3) (dagga, benzene, mandrax, injected drug, other)	0.12	with transactional sex
	alcohol use	continuous	the audit scale, 12 items (25); actual responses ranged from 0–27	0.2	
	transactional sex score	continuous	scores ranged 0–6; based six questions which asked about giving or receiving cash or goods/services in exchange for sex with any of a main partners, once off or khwapheni (secret on-going partner); each assessment was based on 8 possible transacted items	0.3	with lifetime partner numbers
	number of partners (truncated at 50)	continuous	score for partner numbers based on the summed response to six items which asked about numebr of past year main partenrs, once off partners and knwapheni and the same for 'ever'; range was from 0–105, but truncated at 50	0.15	
	physical IPV score	continuous	Score based on 5 behaviourally specific items asked about the last year and 5 before the past year; developed from Garcia-Moreno et al 2005; each had never, once, few, many response options (score range 10–35).	0.19	
**Peer pressure resistence**				0.05	
	related to girlfriend	4 level	I am left out if I do not have a girlfriend because all my friends have one; response on a Lickert scale with 4 options	0.33	
	related to sex	4 level	I have to have sex because all my friends are doing it; response on a Lickert scale with 4 options	0.68	
	related to multiple partners	4 level	I am under pressure to have many partners because all my friends do; response on a Lickert scale with 4 options	0.48	

* vegetarian was a response option but no one endorsed it

In the multinomial regression models we tested a measure of community cohesion, assessed in a five item scale developed for the study. A typical question is “In this area do most people generally trust each other in matters of lending and borrowing?” (Cronbach’s alpha 0.65). We used a scale for peer pressure resistance had a Cronbach’s alpha of 0.72 and for alcohol we used the AUDIT scale with the a cut point of 8 for ‘problem drinking’ [22]. For lifetime number of partners we used a dichotomous variable with a cut point of <8 and 8+ lifetime sexual partners (the 75 percentile). This was for ease of handling the variable. A previous analysis of the dataset showed a dose response relationship between lifetime number of partners and sexual IPV or non-partner rape perpetration [3]. Transactional sex was used as an ever/never binary variable [23, 24]. A gender attitudes and relationship control scale (13-items, Cronbach’s alpha = 0.69) included questions on men’s controlling practices within a relationship and attitudes towards gender relations.

### Ethics

Ethics approval for the study, including consent procedures, was given by the University of Pretoria’s Faculty of Health Sciences Ethics Committee. Permission to work in each community was sought through a community meeting engaging local leaders and community members. All procedures were explained at the community meeting. Participants were mostly recruited in schools, given a leaflet and asked to discuss study participation at home before being enrolled. At enrolment, they signed informed consent and were assured confidentiality. All were offered psychological or other support from a study via a 24 hour cell phone line and given R 20 (then about $2.50) after each interview. At the end of the study the database linking men’s details to their interviews was destroyed. Questionnaires did not collect information that could have enabled an admission of rape perpetration to be linked (by a third party) to a specific act of rape and so disclosure did not pose a risk to study participants. Interviewers were trained in non-collusive interviewing so as not to appear to endorse or minimise rape during the interview questioning [25]. In keeping with South African law at the time of the research pparental consent was not required by the ethics committee for research with minors aged 16 years and over. We discovered that a small number of 15 year olds had been in the study at baseline but had provided misleading information about their age, but we did not learn this until they were 16 (discussed in [20]).

### Data analysis

#### Statistical analysis

Analyses were carried out using Stata 13.0. First descriptive analyses were carried out on the baseline data and variables summarised as percentages or means with 95% confidence limits. Proportions were compared using Pearson’s chi squared test. Means of continuous variables were compared using multinomial regression. Estimates were carried out using standard methods for estimating standard errors from complex multistage sample surveys (Taylor linearization). To fit a multivariable model to investigate the association between the rape categories and variables describing the men’s characteristics, multinomial regression was used with never having raped as the baseline. All variables presented in Table 1 were entered as candidates for the model but we used backwards elimination and retained those with p< or = 0.05. The final model included variables for age and stratum.

#### SEM

Structural Equation Modeling (SEM) was conducted on the baseline data using Stata 13.0 to assess the interrelationship between variables associated with rape in the multinomial regression model. The model outcome was a rape latent variable which is indicated by a continuous measure of sexual intimate partner violence, a binary variable for ever having engaged in multiple perpetrator rape and a binary variable for having ever engaged in a non-partner rape (see Table 1). For model building we prepared each latent variable (see Table 1) and used confirmatory factor analysis to optimise the indicators of each latent variable. The standardized coefficients were examined. Where factor loadings were weak (<0.3), variables were dropped to optimize measurement. The goodness of fit was tested for each latent variable, suggested modification indices were examined and those that fitted theoretically were included such that the fit was optimised through errors being allowed to co-vary. The correlation between each hypothesized latent variable and the rape latent variable were then tested by building variable pairs. All associations were tested by running a full-information maximum likelihood method with adjustment for missing values.

As a next stage, a measurement model was fitted with the latent variables allowed to freely correlate. To assess model fit of the observed data, we used the comparative fit index (CFI) (>0.95); Tucker-Lewis Index (TLI) (>0.9) for acceptable fit and (>0.95) as indicative of good fit [26]; and root mean square error of approximation (RMSEA) (of 0.05 or less) [27, 28]. The model chi-square test was examined, but it was not used in assessing model fit because it has unsatisfactory properties, such as inflation with large sample sizes [28].

**Fig 1:**
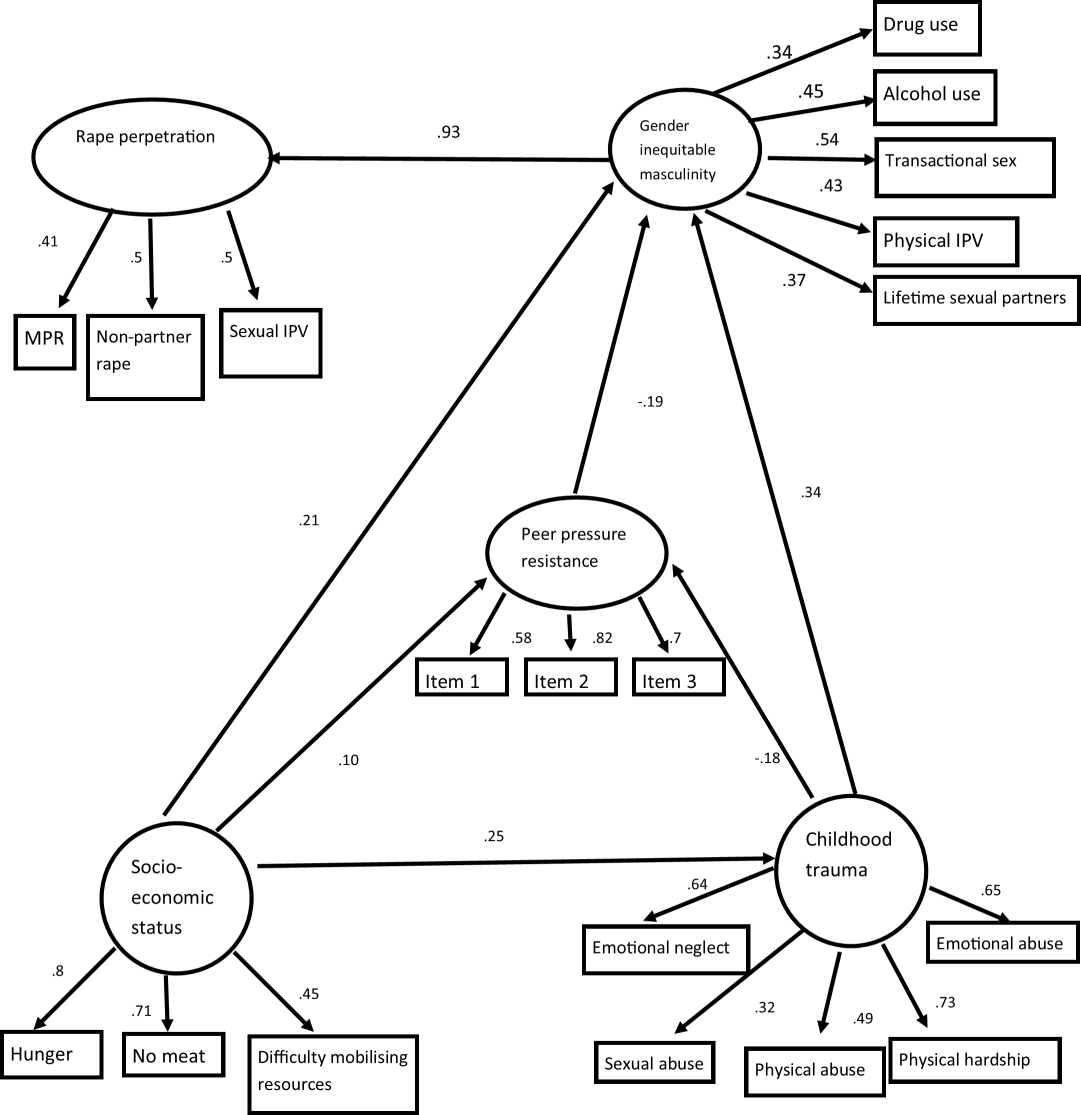
Structural equation model of rape perpetration.

The hypothesized measurement model for the SEM analysis (not shown) was a poor fit of the data and in order to develop a useful and parsimonious model we systematically deleted non-significant paths. Next the structural relations among our latent variables were examined. The final structural model is presented in Fig 1, with the significance of individual paths and final factor loadings indicated; χ^2^ (315.043, degrees of freedom = 136), p < .0001; CFI = .953; TLI = .941; RMSEA = .031. The included correlated errors are indicated in Table 1.

## Results

Among the men in the study 76.1% had never raped, 10.0% had raped as a single perpetrator (SPR) and 13.9% had ever engaged in multiple perpetrator rape (gang rape/MPR). The mean age was 19.1. Only 12.7% had completed Grade 10 or were in a higher school grade. The three groups of men differed in socio-economic status, and in the proportion whose mothers had completed school, with increasing SES and maternal education across groups such that the men who reported MPR were the wealthiest and had the most educated mothers (p<0.0001) (see Table 2). The groups of men also differed significantly in their likelihood of having ever earned money, with more of those who raped as single perpetrators having done so and fewest of those who never raped.

**Table 2:** Socio-demographic and behavioural characteristics of men by rape perpetration category.

	Never raped			Single perpetrator			Multiple perpetrator			
	n = 1039			n = 137			n = 190			
	% / mean	95%CI		% / mean	95%CI		% / mean	95%CI		p value
**Social characteristics**										
Age (years)	19.14	18.96	19.33	19.35*	18.97	19.72	19.00**	18.70	19.29	*0.52 **0.32
Education: over grade 10	12.0	8.6	15.5	16.1	9.0	23.1	14.2	7.4	21.0	0.360
Socio-economic status(mean)	-0.09	-0.23	0.05	0.21*	-0.08	0.49	0.32**	0.05	0.59	*0.05 **0.002
Mother completed school	62.6	58.6	66.7	73.7	65.0	82.5	80.0	73.7	86.3	<0.0001
Ever earned money	50.8	46.7	54.9	71.5	63.3	79.7	63.7	57.1	70.3	<0.0001
**Childhood**										
Sexual abuse in childhood: none	87.6			74.5			66.3			<0.0001
1	9.9			21.2			22.6			
>1 time	2.5			4.4			11.1			
Sexually coerced by a woman	6.3	4.6	7.9	20.4	13.1	27.7	20.0	14.3	25.7	<0.0001
Physical abuse in childhood: score	4.78	4.70	4.87	5.07*	4.89	5.26	4.99**	4.81	5.18	*0.01 **0.09
**Social capital**										
Community cohesion score (high is less cohesive)	-0.06	-0.12	0.01	0.19*	0.00	0.39	0.15**	-0.01	0.31	*0.03 **0.02
Involvement in 1+ clubs	72.5	68.2	76.7	73.0	65.3	80.7	83.2	77.0	89.3	0.007
**Social and peer practices**										
Gang membership & arrests: none	90.1			75.3			77.9			<0.0001
Gang member	2.7			9.9			10.5			
Arrested	5.8			8.6			6.3			
Both gang member & arrested	1.5			6.2			5.3			
Peer pressure resistance	0.06	-0.02	0.14	-0.11*	-0.29	0.06	-0.24**	-0.41	-0.071	*0.09 **0.001
Problem drinking	20.2	17.3	23.1	44.5	35.0	54.0	38.9	31.6	46.3	<0.0001
Ever drug use	34.2	30.1	38.2	52.6	43.0	62.1	51.6	43.3	59.8	<0.0001
**Attitudes and practices of gender relations**										
Gender attitudes and relationship control score	-0.02	-0.13	0.09	0.02*	-0.15	0.20	0.08**	-0.08	0.24	*0.30 **0.26
Any transactional sex	22.0	18.4	25.6	43.4	33.9	52.9	50.8	42.7	58.9	<0.0001
8+ lifetime partners	21.6	18.8	24.3	36.3	26.6	46.0	54.0	46.6	61.3	<0.0001
Ever physical IPV	22.4	19.4	25.3	44.8	35.8	53.8	46.0	39.1	52.8	<0.0001

*comparison of single perpetrator rape and having never raped

** comparison of multiple perpetrator rape and having never raped

Men who raped had experienced more childhood trauma than non-perpetrators. Sexual abuse in childhood was more common among men reporting SPR, and most common among men report MPR, among whom a third had experienced sexual abuse. One in five of the men who had raped had been sexually coerced by a woman, this did not differ between SPR and MPR. Physical punishment, particularly harsher punishment, was almost ubiquitous among the men with only 5.6% reporting none. Exposure to physical abuse and emotional neglect did not differ significantly between the rape categories. Physical hardship and emotional hardship were more common among those who had raped.

The men who had engaged in MPR were significantly more likely to report club or group membership in the community than the other men, and also gang membership. Relatedly men who raped and particularly those reporting MPR were much less resistant to peer pressure (p<0.0001). Men who had raped were more likely to have been arrested (held overnight in a police cell or imprisoned). Substance abuse differed among men who raped compared to those who had not, but not between SPR and MPR. Problem drinking and drug use were much more common among men who had raped.

The three groups of men did not differ in their gender attitudes and reported control of their current partner, but they did in other aspects of sexual practices. The men who raped were much more likely to have had transactional sex and more lifetime partners, with the proportion highest among those reporting MPR. Physical intimate partner violence was much more common among men who raped than others, but the two rape groups did not differ in this regard.

Table 3 shows the multinomial regression model, with the two rape categories compared to never having raped. Compared to having never raped, SPR was associated with sexual abuse in childhood (RRR for one episode 1.93 (95%CI 1.19, 3.11), having been sexually coerced by a woman RRR 2.59 (95%CI 1.44, 4.65), ever having used drugs RRR 1.80 (95%CI 1.17, 2.79), having had transactional sex RRR 1.71 (95% CI 1.06, 2.75), problem drinking RRR 2.11 (95%CI 1.36, 3.27), and having been physically violent towards a partner RRR 1.85 (95% CI 1.19, 2.89).

**Table 3:** Multivariable multinomial regression model of multiple perpetrator rape compared with never having raped and single perpetrator rape.

	Multiple perpetrator rape compared to never having raped				Single perpetrator rape compared to never having raped			
	RRR	95% CI		p value	RRR	95% CI		p value
Socio-economic status scale	1.15	1.00	1.32	0.043	1.11	0.95	1.29	0.201
Sexual abuse in childhood: none	1.00				1.00			
once	1.81	1.16	2.82	0.01	1.93	1.19	3.11	0.008
>1 time	2.86	1.54	5.31	0.001	1.06	0.35	3.20	0.922
Sexually coerced by a woman	1.95	1.19	3.22	0.009	2.59	1.44	4.65	0.002
Peer pressure resistance	0.80	0.67	0.95	0.011	0.92	0.75	1.12	0.394
Gang membership	2.31	1.24	4.31	0.009	1.61	0.82	3.16	0.164
Ever drug use	1.76	1.20	2.57	0.004	1.80	1.17	2.79	0.009
Any transactional sex	2.05	1.35	3.14	0.001	1.71	1.06	2.75	0.027
Alcohol abuse	1.30	0.91	1.85	0.149	2.11	1.36	3.27	0.001
8+ lifetime partners	2.85	1.89	4.28	<0.0001	1.25	0.75	2.09	0.386
Ever physical IPV	1.92	1.37	2.71	<0.0001	1.85	1.19	2.89	0.007

Comparing having never perpetrated rape to having engaged in multiple perpetrator rape, MPR was associated with higher socio-economic status RRR 1.15 (95%CI 1.00–1.32) and having been sexually abused in childhood, with sexual abuse having a dose response relationship, with the relative risk ratios 1.81 and 2.86 for one and more than one episode of sexual abuse respectively. It was also associated with men having been sexually coerced by a woman RRR 1.95 (95% CI 1.19, 3.22). It was associated with a greater susceptibility to peer pressure and a higher likelihood of gang membership RRR 2.31 (95% CI 1.24, 4.31). It was associated with having ever used drugs RRR 1.76 (95%CI 1.20, 2.57) and having had transactional sex with a woman RRR 2.05 (95%CI 1.35, 3.14). It was associated with having had more lifetime partners RRR 2.85 (95%CI 1.89, 4.28) and having been physically violent towards a partner RRR 1.92 (95%CI 1.37, 2.71).

### Structural equation modelling

The structural equation model (presented in Fig 1 and Table 4) shows that there is no direct pathway between socio-economic status and rape perpetration. Paths from all variables impacting rape perpetration were ultimately mediated by gender inequitable masculinity. Socio-economic status had a direct path to gender inequitable masculinity, with the direction of the coefficient indicating that having a higher socio-economic status was correlated with the greater expression of gender inequitable masculine behaviour. It also had two indirect pathways, one mediated by childhood trauma exposure and the coefficient directionality indicated that lower socio-economic status was more strongly correlated with experience of trauma in childhood and thence more likely adopt behaviours indicative of a more inequitable masculinity. Experience of childhood trauma’s impact on gender inequitable masculinity was also mediated by (lesser) peer pressure resistance. There was also an indirect path from socio-economic status to gender inequitable masculinity mediated by peer pressure resistance, with the directionality of the coefficients showing that having a higher socio-economic status, correlated with greater resistance to peer pressure and thence a lesser expression of gender inequitable masculinity. The standardised and unstandardised coefficients, standard errors, p values and direct and indirect effects, as well as R^2^ are presented in Table 4. We tested the model with the svy command, and tested for a moderating effect of age and stratum. None of this improved model fit and so in the interest of parsimony (and a desire to be able to report model fit statistics as these cannot be generated with the svy command) we have not presented this.

**Table 4:** Results of the structural equation modelling analysis.

			Unstandardised coefficient	Standard Error	p value	Standardised coefficient	Standard Error	p value
DIRECT EFFECTS								
Resistence to peer pressure	<--	Childhood trauma	-0.196	0.053	<0.0001	-0.178	0.037	<0.0001
	<--	socio-economic status	0.096	0.043	0.026	0.097	0.037	0.008
Gender inequitable masculinity	<--	Resistence to peer pressure	-0.171	0.051	0.001	-0.190	0.039	<0.0001
	<--	Childhood trauma	0.339	0.056	<0.0001	0.343	0.041	<0.0001
	<--	socio-economic status	0.184	0.051	<0.0001	0.207	0.043	<0.0001
Childhood trauma	<--	socio-economic status	-0.228	0.052	<0.0001	-0.253	0.038	<0.0001
Rape	<--	Gender inequitable masculinity	0.221	0.021	<0.0001	0.920	0.047	<0.0001
INDIRECT EFFECTS								
Resistence to peer pressure	<--	Childhood trauma	0	(no path)				
	<--	socio-economic status	0.045	0.014	0.002	0.045		
Gender inequitable masculinity	<--	Resistence to peer pressure	0	(no path)				
	<--	Childhood trauma	0.034	0.009	<0.0001	0.034		
	<--	socio-economic status	-0.102	0.027	<0.0001	-0.114		
Childhood trauma	<--	socio-economic status	0	(no path)				
Rape	<--	Gender inequitable masculinity	0	(no path)				
	<--	Resistence to peer pressure	-0.038	0.11	0.001	-0.175		
	<--	Childhood trauma	0.083	0.013		0.035		
	<--	socio-economic status	0.018	0.008	0.026	0.085		
TOTAL EFFECTS								
Resistence to peer pressure	<--	Childhood trauma	-0.196	0.053	<0.0001	-0.178		
	<--	socio-economic status	0.14	0.048	0.003	0.142		
Gender inequitable masculinity	<--	Resistence to peer pressure	-0.171	0.051	0.001	-0.190		
	<--	Childhood trauma	0.373	0.059	<0.0001	0.377		
	<--	socio-economic status	0.083	0.037	0.025	0.093		
Childhood trauma	<--	socio-economic status	-0.228	0.052	<0.0001	-0.253		
Rape	<--	Gender inequitable masculinity	0.221	0.021	<0.0001	0.920		
	<--	Resistence to peer pressure	-0.038	0.011	0.001	-0.175		
	<--	Childhood trauma	0.83	0.013	<0.0001	0.346		
	<--	socio-economic status	0.018	0.008	0.026	0.085		
		**R2**						
Resistence to peer pressure		0.050						
Childhood trauma		0.064						
Gender inequitable masculinity		0.176						
Rape		0.846						
**Overall**		0.860						

## Discussion

We have shown that single-perpetrator rape and multiple perpetrator rape share many etiologic factors. The structural equation model (SEM) has rather elegantly enabled us to view the connections between almost the same set of variables that are associated in the multinomial model. It assists in understanding the apparently contradictory findings of research which indicate both social advantage (wealth and status) and social marginalisation as associated with raping. It also shows the behaviours that are often described as isolated sexual practices in the literature [7, 10], but have argued to be connected male behaviours indicative of gender in equitable masculinity [5], do load together on a latent variable (including drug use which is often associated with hypermasculinity) and mediate all the pathways between socio-economic status and raping. This provided quantitative empirical support for the argument that these practices are connected and indicative of an underlying construct which we call gender inequitable masculinity. The model also highlights how childhood trauma exposure impacts rape and the role of peer pressure resistance (and the lack of it).

The multinomial model showed that many factors were associated both with having committed SPR, and MPR, in the case of all the variables that were only associated with one of these types of rape the direction of effect was the same as for the other type and it seems likely that with a larger sample size there would be little difference between the two types. The multinomial model shows some accentuation of the group-related factors of gang membership, and peer pressure resistance were more strongly associated with MPR, and indeed this was a finding of the UN Multi-country Study on Men and Violence [1]. The fact that having more lifetime partners was in the latter category as well, suggests that higher partner numbers may also be substantially a result of peer pressure, rather an a penchant for impersonal sex which has previous been hypothesised by some authors [7].

The analysis confirmed the associated factors shown in previous rape research including sexual abuse in childhood, having engaged in transactional sex and having been physically violent towards an intimate partner. All these were also found in the UN Multi-Country Study on Men and Violence [1]. In the multinomial model the socio-economic status variable showed rape associated with greater economic advantage, although it should be noted that this was relative and not absolute social advantage as the area in which the research was conducted was in general one of rural and urban poverty and there were no wealthy young men in the study. It is interesting that the SEM provided a new layer to this understanding with the finding that in some circumstances it is also correlated with poverty.

The SEM enables an understanding of the apparently contradictory observations that on the one hand rape perpetration, especially multiple perpetrator rape perpetration is classically associated with poverty-stricken areas, such as Philippe Bourgois described in Harlem [1, 11]. But on the other hand, an apparently contradictory observation is that within an impoverished area men who rape are often from more advantaged backgrounds [2, 3, 6]. The model shows that both of these may pertain. So an experience of early privilege and power may directly translate into a configuration of male behaviours which expresses (gender inequitable) masculinised power. On the other hand, experience of socio-economic powerlessness enhances the risk of child abuse and the trauma exposure may in turn result in men seeking to show emphasised power over women, as Bourgois suggests, in response to their own perceptions of their general lack of power [11]. There is a final common pathway, which links the latent variable of gender inequitable masculinity with rape perpetration.

The structural equation model enabled an understanding of how the individual variables associated with rape perpetration should be interpreted. It is important that the variables of partner numbers, transactional sex, physical IPV, drug and alcohol abuse were shown to load well as indicators of an underlying (latent) construct that we refer to as gender inequitable masculinity. The variables of partner numbers, transactional sex, physical IPV have been argued as reflecting an underlying masculinity which is positioned as both dominant over women and showing an emphasised heterosexuality [23, 29]. Drug and alcohol use have been argued to be related to the ‘toughness’ which is viewed as part of hegemonic masculinity (and the hypermasculine extensions) [12]. Previous research has shown that these are very closely associated factors and that the predictors of any one of these largely are associated with the others[3, 30, 31].

The key strength and contribution of this study lies in the fact that the data were collected from a large sample of men, at baseline using particularly few highly trained interviewers. As volunteers, study participants may have differed from young men in the general population, but it is unlikely that this would have made much difference to the estimates of association. There may have been reporting bias but levels of reporting of perpetration were quite high. Although SEM pathways are generally referred to as ‘causal’ the lack of use of longitudinal data in this analysis prevents the interpretation form reflecting true causality.

## Conclusion

This study has shown that the central task of interventions to prevent rape must be to change the socialisation of boys and young men to build more gender equitable ideals of masculinity. The structural context of life in poverty and childhood trauma are key exposures of some men who rape and are important to prevent in their own right. In so-doing, this will contribute to preventing the development of more gender inequitable masculinities. Although social marginalisation has a role in rape prevention, our analysis suggests that removing it on its own will not change the propensity of men to rape women and girls, in order to do so masculinity much be changed.
